# Variability Between Radiation-Induced Cancer Risk Models in Estimating Oncogenic Risk in Intensive Care Unit Patients

**DOI:** 10.3390/tomography11040042

**Published:** 2025-04-03

**Authors:** Emilio Quaia, Chiara Zanon, Riccardo Torchio, Fabrizio Dughiero, Francesca De Monte, Marta Paiusco

**Affiliations:** 1 Department of Radiology, University of Padova, Via Giustiniani 2, 35128 Padova, Italy; chiara.zanon@unipd.it; 2 Department of Information Engineering, University of Padova, 35128 Padova, Italy; riccardo.torchio@unipd.it (R.T.); fabrizio.dughiero@unipd.it (F.D.); 3 Medical Physics Department, Veneto Institute of Oncology IOV—IRCCS, 35128 Padova, Italy; francesca.demonte@iov.veneto.it (F.D.M.); marta.paiusco@iov.veneto.it (M.P.)

**Keywords:** BEIR VII, oncogenic risk, intensive care unit, effective dose, computed tomography, radiation exposure

## Abstract

Purpose: To evaluate the variability of oncogenic risk related to radiation exposure in patients frequently exposed to ionizing radiation for diagnostic purposes, specifically ICU patients, according to different risk models, including the BEIR VII, ICRP 103, and US EPA models. Methods: This was an IRB-approved observational retrospective study. A total of 71 patients (58 male, 13 female; median age, 66 years; interquartile range [IQR], 65–71 years) admitted to the ICU who underwent X-ray examinations between 1 October 2021 and 28 February 2023 were included. For each patient, the cumulative effective dose during a single hospital admission was calculated. Lifetime attributable risk (LAR) was estimated based on the BEIR VII, ICRP 103, and US EPA risk models to calculate additional oncogenic risk related to radiation exposure. The Friedman test for repeated-measures analysis of variance was used to compare risk values between different models. The intraclass correlation coefficient (ICC) was used to assess the consistency of risk values between different models. Results: Different organ, leukemia, and all-cancer risk values estimated according to different oncogenic risk models were significantly different, but the intraclass correlation coefficient revealed a good (>0.75) or even excellent (>0.9) agreement between different risk models. The ICRP 103 model estimated a lower all-cancer (median 69.05 [IQR 30.35–195.37]) and leukemia risk (8.22 [3.02–27.93]) compared to the US EPA (all-cancer: 139.68 [50.51–416.16]; leukemia: 23.34 [3.47–64.37]) and BEIR VII (all-cancer: 162.08 [70.6–371.40]; leukemia: 24.66 [12.9–58.8]) models. Conclusions: Cancer risk values were significantly different between risk models, though inter-model agreement in the consistency of risk values was found to be good, or even excellent.

## 1. Introduction

Medical exposures to ionizing radiation for diagnostic purposes continue to increase [1,2] and the related potential oncogenic effects represent a source of concern [3,4,5]. In radiology, dose metrics are used to optimize the radiation dose to the patient, but they cannot be applied to estimate individual radiation risk based on age, gender, or tissue type. 

To estimate the lifetime risks of radiation-induced cancer, different oncogenic risk models have been developed based on epidemiological data from the Life Span Study [6,7,8,9,10] using the linear no-threshold theoretical principle, which states that any radiation exposure, regardless of dose, increases cancer risk [11]. The National Research Council and National Academy of Sciences published the Biological Effects of Ionizing Radiation seventh report (BEIR VII) in 2006 [12] and proposed a model for estimating radiation-induced oncogenic risk. The BEIR VII model applies a linear-quadratic radiation dose–cancer risk relationship for both acute and chronic radiation exposure and a quadratic relationship for leukemia. The Radiation Risk Assessment Tool [13] is an additional model based on BEIR VII and supported by the National Cancer Institute that was proposed to estimate lifetime cancer risk by integrating life expectancy in the US population and cancer incidence. In addition, the United Nations Scientific Committee on the Effects of Atomic Radiation and the International Commission on Radiological Protection (ICRP) developed two cancer risk models that were presented in the UNSCEAR 2006 report [2] and ICRP Publication 103 [14], respectively. Other risk models that have been developed include the United States Environmental Protection Agency (US EPA) model [15], which represents a developed version of the BEIR VII model, and the World Health Organization model [16], which adapted the latest models of the United Nations Scientific Committee on the Effects of Atomic Radiation and ICRP to conduct off-site analyses of the Fukushima nuclear accident.

A marked reduction in radiation dose is particularly necessary in intensive care unit (ICU) patients, who are frequently exposed to radiation doses as high as 100 mSv or more during a single hospital admission, especially among patients with prolonged hospitalization [5,17]. Due to their clinical status requiring frequent monitoring of medical and/or surgical complications in the chest and/or abdomen, ICU patients frequently undergo CT, often with extended scanning lengths and with the scans repeated several times during a short period of time, resulting in a highly effective dose and increased risk of radiation-induced cancer.

The aim of this study was to evaluate the variability of oncogenic risk related to radiation exposure in patients frequently exposed to ionizing radiation for diagnostic purposes, specifically ICU patients, according to different risk models, including the BEIR VII, ICRP 103, and US EPA models.

## 2. Materials and Methods

### 2.1. Patient Population

This observational single-center retrospective study was conducted in accordance with the Declaration of Helsinki and approved by the hospital IRB, and patient informed consent was obtained (Protocol number 0002146 approved 13 April 2023). We identified all consecutive patients referred to the ICU of our hospital between 1 October 2021 and 28 February 2023 because of their severe clinical status after recent major thoracic or abdominal surgery or solid organ transplant, or major trauma with severe solid organ injuries. Included patients underwent X-ray examinations during their hospitalization in the ICU, such as radiography of the chest, abdomen, limbs, or spine; fluoroscopy and/or interventional radiological procedures; and CT scans of the head, neck, chest, abdomen, pelvis, or limbs, with or without injection of iodinated contrast agents. We excluded pediatric patients (age < 18 years) and patients who had repeated CT scans due to motion artifacts which could lead to the overestimation of CT radiation dose, had a CT scan as part of PET/CT, underwent solely brain CT or limb CT, had a CT scan as part of a research protocol (because scanning protocols could be different from routine CT scans), or had a higher risk of cancer due to genetics, including Li-Fraumeni syndrome, Lynch syndrome, von Hippel–Lindau disease, multiple endocrine neoplasia, and Cowden syndrome.

### 2.2. CT Scanning Protocols

On chest CT, patients were scanned craniocaudally from the lower neck to the level of the costophrenic angle, and on abdominal CT, they were scanned from the diaphragm level to the pelvis, both before and after the injection of iodinated contrast agent. The arterial phase was triggered by placing a region-of-interest over the abdominal CT scan at the level of the second lumbar vertebral body and starting the scan when the density level reached 100 HU. The portal venous and late phases were obtained 70 s and 180 s after iodinated contrast injection, respectively. The following CT parameters were used: tube voltage, 100–120 kVp; automatic tube current modulation activated; gantry rotation period, 280 ms; detector collimation, 0.625 mm; and detector pitch, 1.53. The CT dataset was then reconstructed at a section thickness of 1.25 mm with 512 × 512 matrices using standard kernels for soft tissues.

### 2.3. Cumulative Effective Dose

The cumulative effective dose per patient was calculated by summing the effective dose from each radiological procedure in the ICU during the hospital admission period. The effective dose from the radiography of different body segments was estimated based on median values derived from the literature based on effective dose expressed as organ-absorbed dose in the ICRP reference phantom [17]. To calculate the effective dose for fluoroscopy and interventional radiology procedures, the dose-area product (DAP, expressed in mGy × cm^2^) was multiplied for established conversion factors (k) [17,18,19] expressed in mSv/(mGy × cm^2^) to obtain the effective dose in mSv according to the following formula: effective dose = k × DAP. To calculate the CT effective dose, a Monte Carlo simulation algorithm was used. CT data were deidentified at the facility where they were obtained and uploaded to a single server using *Radimetrics* (Radimetrics Enterprise Platform, Bayer Healthcare, Berlin, Germany), a software tool for monitoring and tracking patient radiation exposures. Two experts in medical physics performed the analysis and provided the final data from *Radimetrics*. The software extracts patient sex, age, date and time of the examination, scan region, study description, protocol name, scanner manufacturer and model, and dose metrics from the Radiation Dose Structured Report (RDSR) sent to the Picture Archiving and Communication Systems (PACS) [19,20]. *Radimetrics* calculates the volume CT dose index (CTDI_vol_), which represents the average radiation exposure per section; the dose-length product, which is the product of CTDI_vol_ and the scan length, measured in mGy × cm; and the effective dose, measured in mSv. To calculate the effective dose, *Radimetrics* matches patients to a particular phantom in its library based on age, weight, or patient diameter and runs a set of Monte Carlo simulations based on the examination parameters. Organ-absorbed doses are used to calculate the effective dose according to tissue-weighting factors defined in ICRP 103 [20].

Data were collected for each irradiation event and then combined and reported according to the examination. We did not include scout scans and bolus-track scans obtained to determine the timing of the injection of iodinated contrast material.

### 2.4. Radiation-Induced Oncogenic Risk

The main features of the radiation dose–cancer risk models are reported in Table 1. In this study, we included the BEIR VII, ICRP 103, and US EPA risk models. We did not include the Radiation Risk Assessment Tool due to its similarity to the BEIR VII model or the United Nations Scientific Committee on the Effects of Atomic Radiation model due to a lack of specific recommendations regarding the risk reduction at low doses and low dose rates [12,13,14]. All risk models included a lifetime attributable risk (LAR), which represents an approximation of the risk of radiation-induced death, defined as death or incident cases of cancer that would have occurred without radiation exposure but occurred at a younger age because of radiation exposure, and that corresponds to the probability that an individual will die from or develop cancer associated with radiation exposure according to sex-, age-, and tumor site-specific coefficients. LAR is expressed as the number of excess cancers or deaths due to cancer per 100,000 persons of mixed ages exposed to 0.1 Gy [12]:

$$LAR\left(D,\;e\right)=\int_{e+L}^{amax}M\left(D,e,a\right)\;S(a)/S(e)\;da$$

LAR is mathematically defined as the sum of risks between the age at exposure (*e*) plus a latency period (*L*), which is set at 5 years for solid cancers and 2 years for leukemia based on evidence from the Life Span Study, and a maximum age (*amax*), set at 100 years.  M (D, e, a) corresponds to the absolute risk at attained age *a* from an exposure at age *e*, *S_(a)_* represents the probability of surviving to age *a* (cancer-free survival), and *S_(a)_*/*S_(e)_* represents the conditional probability of a person being alive and cancer-free at age *e*, reaching at least an attained age *a*. LAR is defined within an interval of 10 years for each age class for individuals aged >18 years, as considered in this study. For individuals included in a different age class, the LAR risk was calculated by using linear interpolation. For example, for an individual aged 75 years, the risk was calculated by linear interpolation between 70 and 80 years.

To express the radiation-induced cancer risk, different from the other risk models, ICRP 103 [15] includes the risk of exposure-induced cancer incidence, which can be approximated to LAR for low-dose exposure and is calculated as follows:

$$\int_{e+L}^{a_{max}}[\mu_{ic}\left(a|D,e\right)-\mu_{ic}(a)]\cdot S\left(a|D,e\right)da$$

where (*a|D, e*) and μ_ic_(*a*) indicate the specific cancer incidence (*c*) at age *a* with or without radiation exposure, *L* represents the minimal latency period, and *S (a|D, e)* indicates the probability of cancer-free survival.

One expert in mathematics performed the risk analysis. To obtain comparable numbers between different risk models, the oncogenic risk was expressed as the additional oncogenic risk (AOR)—the risk index of cancers in patients exposed to X-rays in addition to the oncogenic risk in the general population. AOR is calculated based on the linear no-threshold model. AOR corresponds to the LAR per 100,000 individuals per 0.1 Gy multiplied by cumulative effective dose/0.1 in the BEIR VII model, and to the LAR per 10,000 individuals per Gy multiplied by cumulative effective dose in the ICRP 103 and US EPA risk models [21,22]. To make numbers comparable, AOR was finally multiplied by 100,000.

### 2.5. Statistical Analysis

All anonymized data were exported to an Excel file (Microsoft Office Professional Plus 2010, Microsoft Corporation, Redmond, WA, USA). Statistical analyses were performed using MedCalc for Windows, v. 23.1.3 (MedCalc Software, Mariakerke, Belgium). We estimated needing to recruit at least 50 patients to detect a significant difference in cancer risk considering a statistical power of 0.8 and significance criterion of 0.05. For all analyses, orthogonal regression was conducted, and the Pearson r was computed. To evaluate consistency, intraclass correlation coefficients (ICCs) were determined and Bland–Altman analyses were performed. We used the ICC to assess the consistency of risk values between different models. ICC values < 0.5 indicate poor agreement; values between ≥0.5 and <0.75 indicate moderate agreement; between ≥0.75 and <0.9, good agreement; and ≥0.9, excellent agreement. Results for continuous variables were expressed as the median and interquartile range (IQR). As the Shapiro–Wilk test failed to show a normal data distribution, the Friedman test for repeated-measures analysis of variance was used to compare risk values between different models. *p* < 0.05 was considered indicative of a difference.

## 3. Results

### 3.1. Patient Characteristics

**Figure 1:**
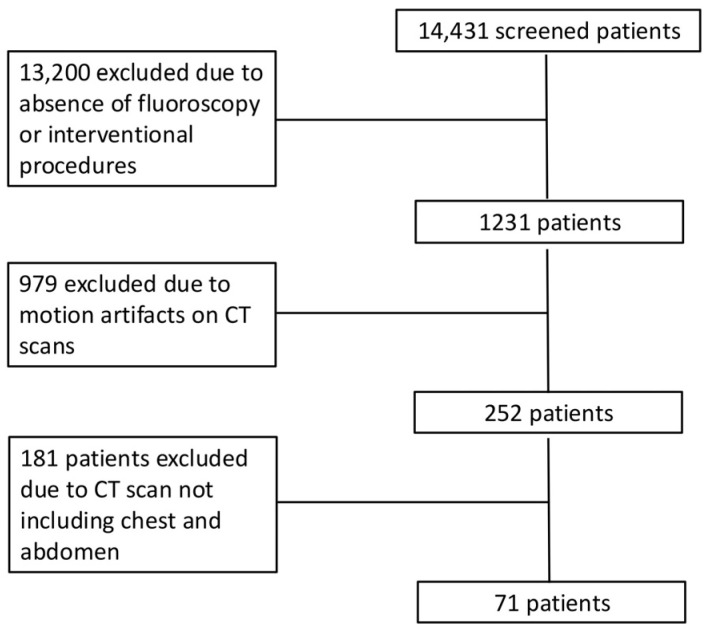
Patient flow chart.

The inclusion process for patients in the study is shown in Figure 1. After exclusion, we included 71 patients (Table 2) who underwent 2847 radiological examinations, including 2472 X-ray films (87%), 270 contrast-enhanced CT scans (9%), and 106 fluoroscopy and interventional radiology procedures (4%). Patients underwent CT of different body regions: 19 underwent chest CT (n = 10 unenhanced CT and after iodinated contrast injection during the arterial phase, n = 9 unenhanced CT and contrast-enhanced CT in both arterial and portal venous phases), 20 abdominal CT (n = 12 unenhanced CT and after iodinated contrast injection during the arterial phase, n = 8 unenhanced CT and contrast-enhanced CT in both arterial and portal venous phases), and 32 chest and abdominal CT (n = 12 unenhanced CT in arterial phase, n = 20 unenhanced CT in both arterial and portal venous phases).

### 3.2. Cumulative Effective Dose

**Figure 2:**
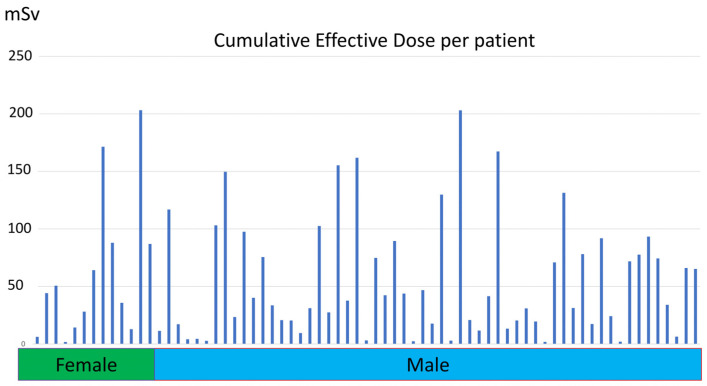
Histogram shows cumulative effective dose distribution according to patient sex.

Figure 2 shows the cumulative effective dose distribution according to sex. The median cumulative effective dose per patient was 38.82 (IQR, 17.34–84.64) mSv. Male patients were exposed to a higher median cumulative effective dose than female patients (37.59 [IQR, 17.6–78.01] vs. 44.11 [IQR, 14.19–86.85]).

### 3.3. Oncogenic Risk

**Figure 3:**
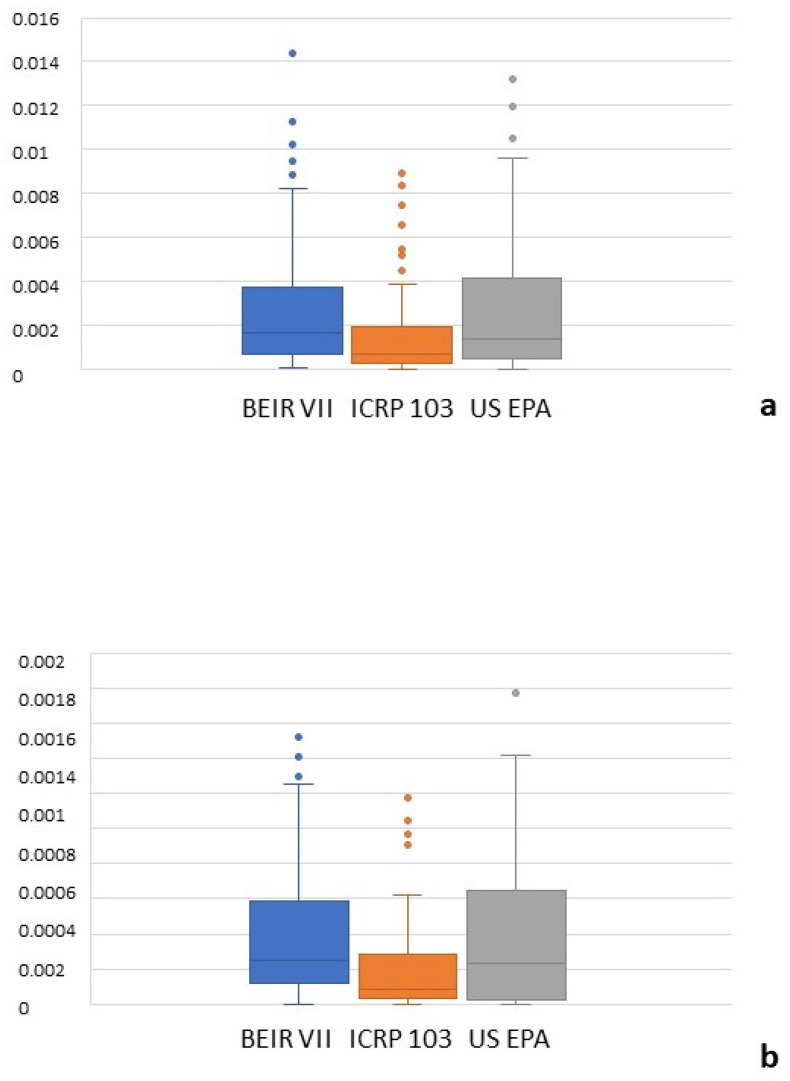
Box plots showing the all-cancer risk (**a**) and leukemia risk (**b**) distribution according to the oncogenic risk model. The box includes the first and third quartile, and the horizontal line corresponds to the median value. Vertical lines extending from the box indicate variability outside the upper and lower quartiles with whiskers indicating the maximum and minimum value. Outliers are plotted as individual points beyond the whiskers.

**Figure 4:**
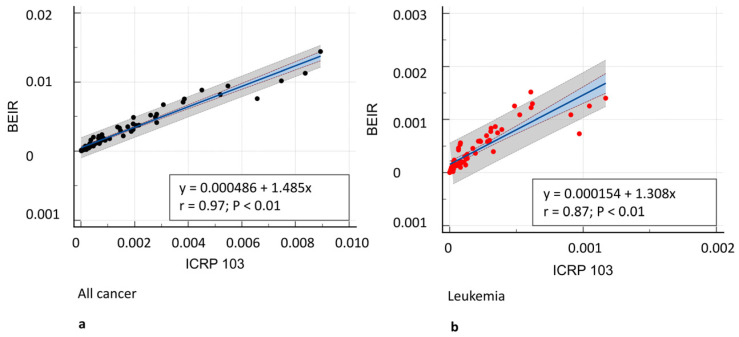

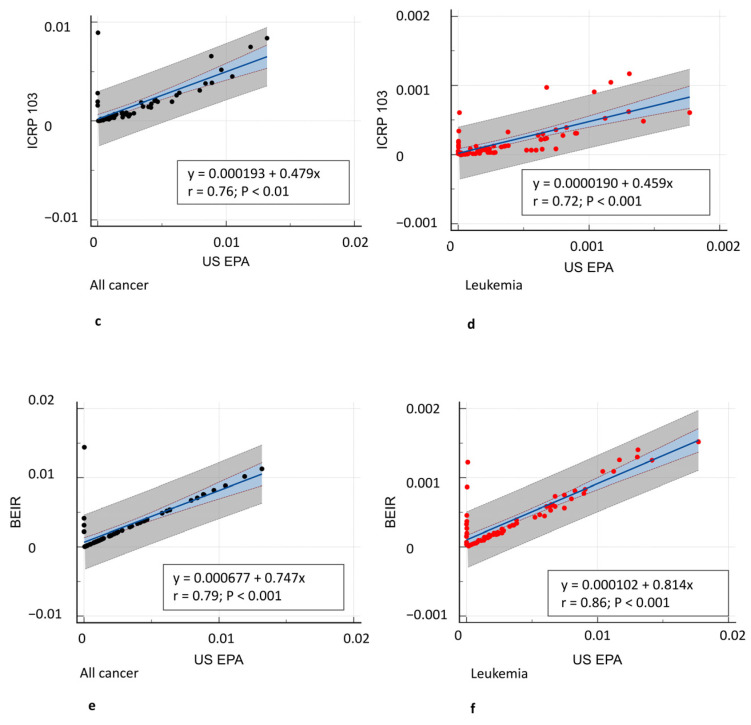
Correlation analysis between different risk models. (**a**) All-cancer risk (black dots) in BEIR VII vs. ICRP 103, (**c**) ICRP 103 vs. US EPA, and (**e**) BEIR VII vs. US EPA risk models. (**b**) Leukemia risk (red dots) in BEIR VII vs. ICRP 103, (**d**) ICRP 103 vs. US EPA, and (**f**) BEIR VII vs. US EPA risk models. A significant correlation was observed between all risk models (*r* = 0.71–0.97, *p* ≤ 0.0001).

**Figure 5:**
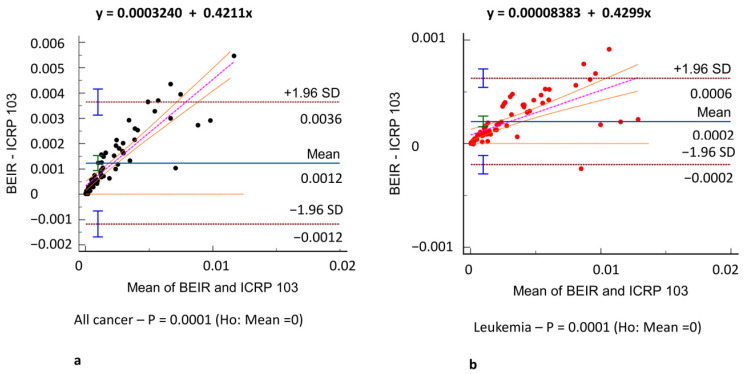

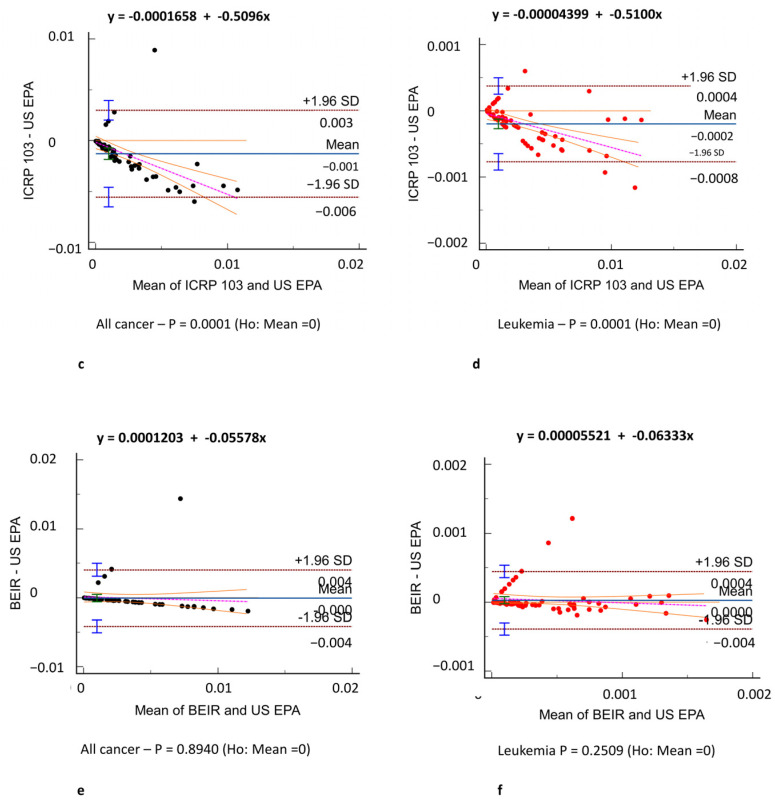
Agreement assessment according to the Bland–Altman analysis between different risk models. (**a**) All-cancer risk (black dots) in BEIR VII vs. ICRP 103, (**c**) ICRP 103 vs. US EPA, and (**e**) BEIR VII vs. US EPA risk models. (**b**) Leukemia risk (red dots) in BEIR VII vs. ICRP 103, (**d**) ICRP 103 vs. US EPA, and (**f**) BEIR VII vs. US EPA risk models. The difference in the estimated risks is on the *y*-axis and the mean of the two model estimated risks is on the *x*-axis. Comparisons revealed very low bias (mean difference: −0.001–0.004) in both all-cancer (**e**) and leukemia risk (**f**) agreement.

Figure 3 shows the all-cancer and leukemia risk distribution according to different risk models. A significant correlation was observed between all risk models (r = 0.71–0.97, *p* = 0.001) (Figure 4) and Bland–Altman analysis showed negligible bias (mean difference, −0.001–0.004) (Figure 5). Different organ, leukemia, and all-cancer risk values (Table 3) were significantly different, but the ICC revealed good or even excellent inter-model consistency in risk values. The ICRP 103 model indicated a lower all-cancer and leukemia risk compared to the US EPA and BEIR VII models (Table 3). Both the BEIR VII and ICRP 103 models indicated higher all-cancer risk in females, whereas the US EPA model showed higher all-cancer risk in males (Table 4). ICRP 103 showed a higher leukemia risk in females, whereas BEIR VII showed a higher leukemia risk in males (Table 4).

## 4. Discussion

In this study, we compared the calculated oncogenic risk related to radiation exposure across a clinical dataset according to the most accredited risk models. As effective dose cannot express the individual oncogenic risk, factors such as sex, age, and tissue characteristics affecting individual radiation risk have been proposed as surrogates for actual patient risk [23]. To make numerical values obtained by different risk models comparable, the oncogenic risk was expressed as AOR, calculated from the effective dose and LAR product, and corresponding to the risk index of cancers in patients exposed to X-rays in addition to the oncogenic risk in the general population.

The first result of our study was that different organ, leukemia, and all-cancer risk values estimated according to different oncogenic risk models were significantly different in patients with frequent exposure to ionizing radiation for diagnostic purposes, such as ICU patients. Yet, the ICC revealed good or even excellent inter-model consistency in risk values. The evidence of significantly different oncogenic risk values should imply the need for more reliable and reproducible cancer risk models based on recent epidemiological data in addition to the epidemiological data from atomic bomb survivors in the Life Span Study [6,7,8,9,10], who had whole-body exposure to X-rays and gamma-rays. In particular, the lower all-cancer and leukemia risks we found when applying the ICRP 103 risk model are likely related to the use of a broader reference population (Europe, USA, and Asia vs. USA population used by all other risk models), a higher excess absolute risk/excess relative risk ratio (0.5/0.5 vs. 0.3/0.7 used by all other risk models), and a higher value for the dose and dose rate effectiveness factor—known as DDREF—(2 vs. 1.5 used by all other risk models). Further parameters, such as differences in ethnicity and diet between the population of our study and the population on which these models were developed, could explain the variability in the risk we obtained. Moreover, estimates by the ICRP 103 and US EPA models were based mainly on mortality data from the Life Span Study from 1950 to 1985 [12], whereas BEIR VII evaluated site-specific cancer mortality data throughout 1997 and cancer incidence data throughout 1998. ICRP 103 estimates are intended to be relevant for the world population, whereas the other estimates were specifically for the US population. The variation in estimates for site-specific cancers further highlights the general uncertainties inherent in this process. Therefore, it would be inappropriate to extrapolate risk factors from the Japanese population at the time of the bombings to a contemporary European or US population, and there is a need to collect recent radiation exposure data to develop a new, epidemiologically validated radiation oncogenic risk model which can address all these limitations.

We found the all-cancer risk to be lower in males than in females according to both the BEIR VII and ICRP 103 models, whereas the leukemia risk was higher in males than in females, according to BEIR VII, and lower in males than in females, according to the ICRP 103 model. Considering the quadratic relationship and 5-year latency for leukemia in all risk models, these results are most likely related to male ICU patients with a higher median age and longer hospital stays and, consequently, a higher cumulative effective dose from radiological procedures, causing a higher risk of radiation-induced solid cancers and leukemia. The higher all-cancer risk found in female patients was likely related to the generally higher radiosensitivity of female patients included in the risk models and related to the higher radiation-related risk in breast, ovary, and uterine cancers.

Several epidemiological studies have suggested that the lowest dose of ionizing radiation at which good evidence of increased cancer risks in humans exists is 10–50 mSv for an acute exposure and 50–100 mSv for prolonged exposure [24,25]. However, whether a threshold exists below which no risk for cancer is present is not clear. Epidemiological data from cohorts of individuals exposed occupationally or cohorts of atomic bomb survivors demonstrate that ionizing radiation is capable of inducing many types of cancer, even at a low-dose exposure [26,27,28,29]. The most supported hypothetical mathematical model of the radiation dose–cancer risk dose–response relationship at low exposure levels is currently the linear no-threshold model [30,31] which represents the theoretical basis of all risk models proposed until now. In the linear no-threshold model, cancer risk caused by ionizing radiation is directly proportional to the amount of radiation exposure to the human body (response linearity), and ionizing radiation is always considered harmful because there is no threshold below which an amount of radiation exposure to the human body is not harmful. Other models, such as the threshold model [30,31] which assumes that very small radiation doses are harmless and provides an hypothesized, but still undefined, threshold radiation dose below which no harmful effects are present; the hypersensitivity model [32], which proposes an increased proportional harmful effect at low dose; and the hormesis model [33,34] which proposes a hypothetical protective effect from ionizing X-ray radiation at low levels, are less supported, especially in vivo. Unfortunately, the linear no-threshold model does not present any epidemiological validation and overstates the risk of radiation-induced carcinogenesis at low doses. No prospective epidemiological studies with appropriate nonirradiated controls have definitively demonstrated the linear no-threshold model, the adverse effects, or the hormetic effects of radiation doses < 100 mSv in humans.

Slovis et al. [35] reported a mean cumulative effective dose ± standard deviation of 22.2 ± 25 mSv from CT examinations among ICU patients, whereas our findings indicate a higher cumulative effective dose from all radiological investigations (median, 38.82 mSv [IQR, 17.34–84.64 mSv]), likely related to the higher clinical complexity of patients included in our dataset who mainly received major surgery or transplant procedures. Consequently, we identified a radiation-induced cancer risk higher than the risk reported by Slovis et al. [35]. Yee et al. [36] indicated an increase in the median cumulative effective dose from 34.59 mSv in 2004 to 40.51 mSv in 2009. Our study confirms that diagnostic imaging with ionizing radiation plays a critical role in diagnosing and managing ICU patients, and generally, in all patients with life-threatening conditions, though radiation exposure should be minimized in this patient category while preserving diagnostic quality. Radiologists should pay specific attention to those CT technical parameters (kV, mA, collimation, X-ray filters, table feed, pitch and automatic exposure control) which influence the radiation effective dose during the acquisition phase. Specific emphasis should be placed on dose-reduction methods, such as ultra-low-dose CT and low-dose scanning and reconstruction techniques based on iterative algorithms or deep learning artificial intelligence. Low-dose [37] and ultra-low-dose CT [38] offering a sub-millisievert radiation should be considered routinely in ICU patients, especially if scanned several times during hospitalization period, to limit radiation exposure. This comes at the cost of increased CT image noise, but at a level which does not hamper the diagnostic content of images. Further advances in technology, including improvements in automatic exposure control and beam-shaping bow-tie filters, could allow tailored adjustments for individual patient anatomy and clinical indications [39,40]. Educational initiatives for healthcare workers to increase awareness about radiation risks and to promote safer practices include the limitation of unjustifiable chest X-rays, fluoroscopy and frequent repetition of CT scans, the employment of real-time dose monitoring systems, and a general, more balanced approach to radiological imaging in ICUs, stressing the need to minimize radiation exposure while ensuring the continuity of high-quality medical care. According to the results of our study, a sustainable ICU radiological practice needs to be developed that merges patient safety, quality care, and optimal use of radiological investigation supported by continuous multidisciplinary research and technological innovation [41].

This study has some limitations due to its retrospective nature and single-center design, which focused on a single hospital admission. Further limitations of our study include the limited number of patients and the lack of clinical verification of our results, which does not allow us to determine which oncogenic risk model is the most reliable.

In conclusion, cancer risk values were significantly different between risk models, though inter-model agreement in the consistency of risk values was found to be good, or even excellent.

## Figures and Tables

**Table 1:** Differences between the different models for estimating lifetime risk of cancer.

	BEIR VII	ICRP 103	US EPA
**Epidemiological data**	***Solid cancer:*** Japanese atomic bomb survivor morbidity data from the period 1958–1998		
	***Leukemia:*** Japanese atomic bomb survivor mortality from the period 1950–2000		
**Reference population**	USA	Europe, USA, Asia	USA
**Excess absolute risk/excess relative risk**	0.3/0.7	0.5/0.5	0.3/0.7
**Dose-response model**	Linear no-threshold		
**Minimal tumoral latency period**	5 years for solid tumors 2 years for leukemia		
**Dose and dose rate effectiveness factor**	1.5	2	1.5
**Lifetime attributable risk**	Weighted geometric mean	Weighted arithmetic mean	Weighted arithmetic mean

Note—BEIR VII = Biological Effects of Ionizing Radiation VII report. ICRP 103 = International Commission on Radiological Protection Publication 103. US EPA = United States Environmental Protection Agency. The dose and dose rate effectiveness factor were proposed to overcome discrepancies between experimental and epidemiological data from high-dose and dose-rate exposure as derived from epidemiological data from the atomic bomb explosion and allows the estimation of risk from low-dose and dose-rate exposures. The dose and dose rate effectiveness factor implies that, for low doses, the probability of DNA damage being carcinogenic is reduced by a factor of 1.5 or 2.

**Table 2:** Patient characteristics.

**Characteristics**	
Age (years)	66 (56–71)
Male	66 (55–71)
Female	61 (58–73)
Sex	
Male	58
Female	13
Hospitalization (days)	31 (15–70)
Male	30 (21–70)
Female	20 (15–26)
Reason for hospitalization	
Major thoracic surgery	19
Major abdominal surgery	27
Solid organ transplant	20
Major trauma	5

Note—values are given as median (interquartile range) or no. of patients.

**Table 3:** Additional oncogenic risk × 100,000 between different risk models for different cancer categories.

Cancer Categories	BEIR VII	ICRP 103	US EPA	*p ^#^*	ICC (95% CI)
*Bladder*	20.75 (8.78–46.89)	7.22 (2.96–20.93)	10.55 (4.07–28.23)	0.0001	0.87 (0.81–0.91)
*Bone*	*	*	0.29 (0.001–1.66)		
*Breast*	10.55 (3.95–27.44)	17.37 (1.63–23.62)	*	0.003	0.9 (0.67–0.97)
*Colon*	25.77 (12.23–66.67)	7.14 (3.09−17.74)	25.69 (12.2–67.85)	0.0001	0.85 (0.79–0.90)
*K* *idney*	*	*	20.45 (9.36–50.41)		
*Liver*	3.6 (1.37–9.24)	2.79 (1.24–7.48)	6.18 (3.19–17.04)	0.0001	0.88 (0.83–0.92)
*Lung*	30.81 (13.43–74)	23.35 (9.75–60.48)	31.06 (13.5–78.79)	0.0001	0.98 (0.97–0.98)
*Esophagus*	*	4 (1.71–8.88)	*		
*Ovary*	6.93 (2.29–13.32)	3.99 (0.69–6.1)	*	0.001	0.7 (0.17–0.91)
*Prostate*	4.37 (0.65–15.26)	*	9.46 (1.39–34.68)	0.001	0.71 (0.58–0.81)
*S* *kin*	*	*	29.43 (11.43–72.64)		
*S* *tomach*	5.26 (2.13–15.27)	4.14 (1.75–13.63)	11.46 (4.93–27.16)	0.001	0.87 (0.81–0.91)
*Thyroid*	0.10 (0.02–0.5)	0.46 (0.35–2.03)	4.57 (1.52–8.71)	0.001	0.38 (0.23–0.53)
*Uterus*	3.50 (1.14–6.78)	*	*		
*Other solid tumors*	28.93 (11.16–71.35)	10.34 (3.09–30.84)	6.43 (2.16–12.17)	0.001	0.9 (0.86–0.93)
*Leukemia*	24.66 (12.9–58.8)	8.22 (3.02–27.93)	23.34 (3.47–64.37)	0.001	0.83 (0.76–0.88)
*All solid tumors*	132.80 (53.76–316.2)	*	86.78 (16.66–296.53)	0.001	0.97 (0.88–0.99)
*All cancers*	162.08 (70.6–371.4)	69.05 (30.35–195.37)	139.68 (50.51–416.16)	0.001	0.91 (0.87–0.94)

Note—additional oncogenic risk (AOR) × 100,000 between the BEIR VII, ICRP 103, and U.S. EPA risk models. Values are median (interquartile range, IQR). * = Not estimated by the model. *^#^* Friedman test repeated-measures analysis of variance. ICC = Intraclass correlation coefficient. CI = Confidence interval.

**Table 4:** Comparison of additional oncogenic risk × 100.000 for all-cancer risk and leukemia risk in each sex according to different risk models.

	BEIR VII	ICRP 103	US EPA	*p ^#^*	ICC (95% CI)
*All-cancer*, *male*	161.86 (72.82–376.95)	65.97 (30.9–187.03)	188.88 (81.73–418.07)	0.001	0.91 (0.87–0.94)
*All-cancer*, *female*	220.46 (746.82–4148.07)	175.69 (48.93–280.85)	60.91 (42–134.19)	0.018	0.44 (0.2–0.94)
*Leukemia*, *male*	29.47 (14.04–65.36)	8.22 (3.09–28.33)	28.36 (14.88–67.68)	0.0001	0.85 (0.79–0.90)
*Leukemia*, *female*	19.87 (68.82–366.38)	11.97 (2.33–18.75)	12.86 (6.11–25.61)	0.00034	0.38 (0.1–0.80)

Note—comparison of Additional Oncogenic Risk (AOR) × 100.000 for all-cancer risk and leukemia-risk according to BEIR VII, ICRP 103 and US EPA risk models in males and females. Values are median (interquartile range, IQR). EPA presents a single estimate for both sexes. *^#^* Friedman test repeated-measures analysis of variance. ICC = Intraclass correlation coefficient. CI = Confidence interval.

## Data Availability

Data are contained within the article.

## Abbreviations

AOR	Additional oncogenic risk
BEIR VII	Biological Effects of Ionizing Radiation seventh report
CTDI_vol_	Volume computed tomography index
ICRP 103	International Commission on Radiological Protection 103
ICC	Intraclass correlation coefficient
ICU	Intensive care unit
IQR	Interquartile range
LAR	Lifetime attributable risk
US EPA	United States Environmental Protection Agency
